# An integrative literature review of kidney transplantation knowledge tools

**DOI:** 10.1371/journal.pone.0281073

**Published:** 2023-01-31

**Authors:** Chan Mi Kang, Hyejin Lee

**Affiliations:** 1 Department of Nursing, Dong-Eui Institute of Technology, Busan, South Korea; 2 Department of Nursing, Dong-Eui University, Busan, South Korea; Medical University of Gdansk, POLAND

## Abstract

**Purpose:**

This study aimed to identify knowledge measurement tools for kidney transplantation (KT) and confirm their assessment methods, domains, and validity to provide useful information.

**Design:**

An integrative review based on Whittemore and Knafl’s (2005) framework and the Preferred Reporting Items for Systematic Reviews and Meta-Analysis (PRISMA) 2020 guidelines.

**Methods:**

An integrative search was conducted using four English databases (PubMed, Embase, CINAHL, and the Cochrane Library) and the top three Korean databases (RISS, DBpia, and KISS). Search terms and strategies included (“kidney transplant*” OR “renal transplant*” OR “kidney replace*” OR “renal replace*” OR “kidney graft” OR “renal graft”) & (knowledge OR awareness) & (scale OR tool OR instrument OR questionnaire OR inventory). The time limit was set to February 2022. The extracted data included the content of the tools, participants, and psychometrics. Quality of life was assessed using a psychometric grading framework.

**Results:**

A total of 15 studies and 13 tools were reviewed. Of these, seven studies (46.7%) targeted KT patients, five (33.3%) targeted KT candidates, and three (20.0%) included both groups. The number of items in the tools ranged from five to 33 items. Furthermore, seven tools comprised true-false questions and eight multiple-choice questions. The domains of the KT knowledge measurement tool used in each study differed across study participants. Both reliability and validity were confirmed in six tools, and only two showed a grade of “adequate” or higher.

**Conclusion:**

A validated tool is required to measure KT knowledge. These tools can be used to evaluate the effectiveness of educational interventions in promoting self-management after KT.

**Protocol registration number:**

CRD42022334559.

## Introduction

The World Health Organization (WHO) reported that there were 100,097 kidney transplantations (KT) worldwide in 2019, 4.8% higher than the previous year [1]. This increase is accompanied by an annually growing number of patients awaiting KT—in Korea, the cumulative number increased from 9,622 in 2010 to a staggering 27,062 in 2020 [2]. As of March 2021, there were approximately 27,142 KT candidates [3].

The growing need for KTs is accompanied by the need for patients to have adequate knowledge on how to manage their kidney transplant, both before and after surgery. This involves knowing how to take immunosuppressants correctly, undertake safe practices and behaviors to prevent infection, and to know of and identify signs or symptoms of a transplant rejection [4–6]. In the pre-transplant phase, knowledge can reduce patients’ fear and confusion [7] and can help them adapt to changes in their healthcare. In the post-transplant phase, knowledge of KT is positively correlated with self-management performance and treatment compliance [4, 8]. Given the significance of knowledge for KT patients, it is imminent that tools to accurately assess and identify gaps in patient knowledge, for nurses to provide appropriate and complete education to KT patients and candidates.

There is a dearth of tools that accurately assess KT knowledge levels. Various studies on KT knowledge have provided educational interventions for KT patients and candidates. Mainly intervention studies, these consisted of educational telephone counseling [9], a survey on the knowledge and treatment adherence of KT patients [10], and a psychometric test of knowledge measurement tools for KT [11]. At present, existing literature does not comprehensively evaluate the reliability and validity of the various types of knowledge measurement tools used in these intervention studies.

### Study aim

This study aimed to identify knowledge measurement tools for KT developed specifically for KT patients and candidates. A secondary aim is to identify the knowledge domains of these tools and evaluate their validity and reliability.

The research questions were as follows:

(1) What measurement methods were used to evaluate KT knowledge in KT patients and candidates?; (2) What knowledge domains do the tools consist of?; (3) How were measurement tools verified?

## Methods

### Design

An integrated literature review was conducted following the PRISMA guidelines (S1 and S2 Tables). As suggested by Whittemore and Knafl [12], the literature was reviewed in order of “research question, literature search, literature evaluation, literature analysis, and presentation of the results.”

### Eligibility criteria

Inclusion criteria for the literature were that the studies: 1) focused on knowledge of KT; 2) included KT patients or candidates (kidney disease, renal failure, peritoneal dialysis, or hemodialysis patients) as participants; 3) were complete reports or had full-text available; and 4) were written in Korean or English. Literature omitted from this review were those that had no explanation of the tools, or that were dissertations, editorials, conference abstracts, or review papers.

### Information sources and search strategy

A literature search was conducted between 10^th^ February and 11^th^ March, 2022 of studies related to knowledge measurement tools for KT patients or candidates. This included literature that had been published in English and Korean journals up till February 2022. Databases searched included four English databases (MEDLINE via PubMed, EMBASE, CINAHL, and Cochrane Library) and three Korean databases (Research Information Sharing Service [RISS], Korean Studies Information Service System [KISS], and Data Base Periodical Information Academic [DBpia]). Medical Subject Headings (MeSH) were used to select keywords, and frequent keywords were identified based on a review of the English titles and keywords. Accordingly, we used the following search strategy (“kidney transplant*” OR “renal transplant*” OR “kidney replace*” OR “renal replace*” OR “kidney graft” OR “renal graft”) AND (“knowledge” OR “awareness”) AND (“scale” OR “tool” OR “instrument” OR “questionnaire” OR “inventory”). Additional studies were identified through citation and reference list searches, and manual searches using Google Scholar.

### Study selection

Two independent researchers selected the literature, and any disagreements and opinions were resolved through sufficient discussion. The retrieved literature was listed, reviewed, and organized using reference management software (EndNote 20.2.1). After removing duplicate studies, the remaining titles and abstracts were reviewed. After this, full texts were reviewed in accordance with the inclusion and exclusion criteria. Final studies were selected for review during researchers’ meetings.

### Data collection process and data items

General characteristics of studies that were included were organized by country, publication year, research design, and study participants. The KT knowledge tools were organized for analysis in the following order: applied participants, scale names, total number of items, domains, score calculation method, score range, total score, and psychometrics, including reliability and validity. Two researchers independently reviewed and integrated the entire literature, and sufficient discussions and reviews were repeated until a common opinion was drawn regarding the discrepancy of items.

### Quality assessment of the included tools

The quality of the measurement tools was evaluated independently by two reviewers using a psychometric grading framework (PGF) [13]. The PGF is based on the most commonly used statistical tests and values recommended by leading psychologists and biostatisticians [13]. It utilizes a grading system of A to D to rate the following aspects of a tool: content, construct, and criterion validity, internal consistency, and test-retest and inter-rater reliabilities. The overall psychometric strength is evaluated by combining the measured number of psychometrics and their grades. Three or more As and/or Bs are rated as good, two As and/or Bs ± C or D as adequate, one A or B ± C or D as weak, and one or more C or D as very weak [13].

### Synthesis

As this review did not intend to compare effects, only studies reporting tools with measurable reliability and validity were assessed for quality, and each tool was presented in an integrative way.

### Ethical considerations

This study was approved by the Institutional Review Board of the University of Dong-Eui Institutional Review Board (DIRB-202203-HR-W-04). This study was approved by PROSPERO. Protocol registration number: CRD42022334559 03/06/2022 (S1 File).

## Results

**Fig 1:**
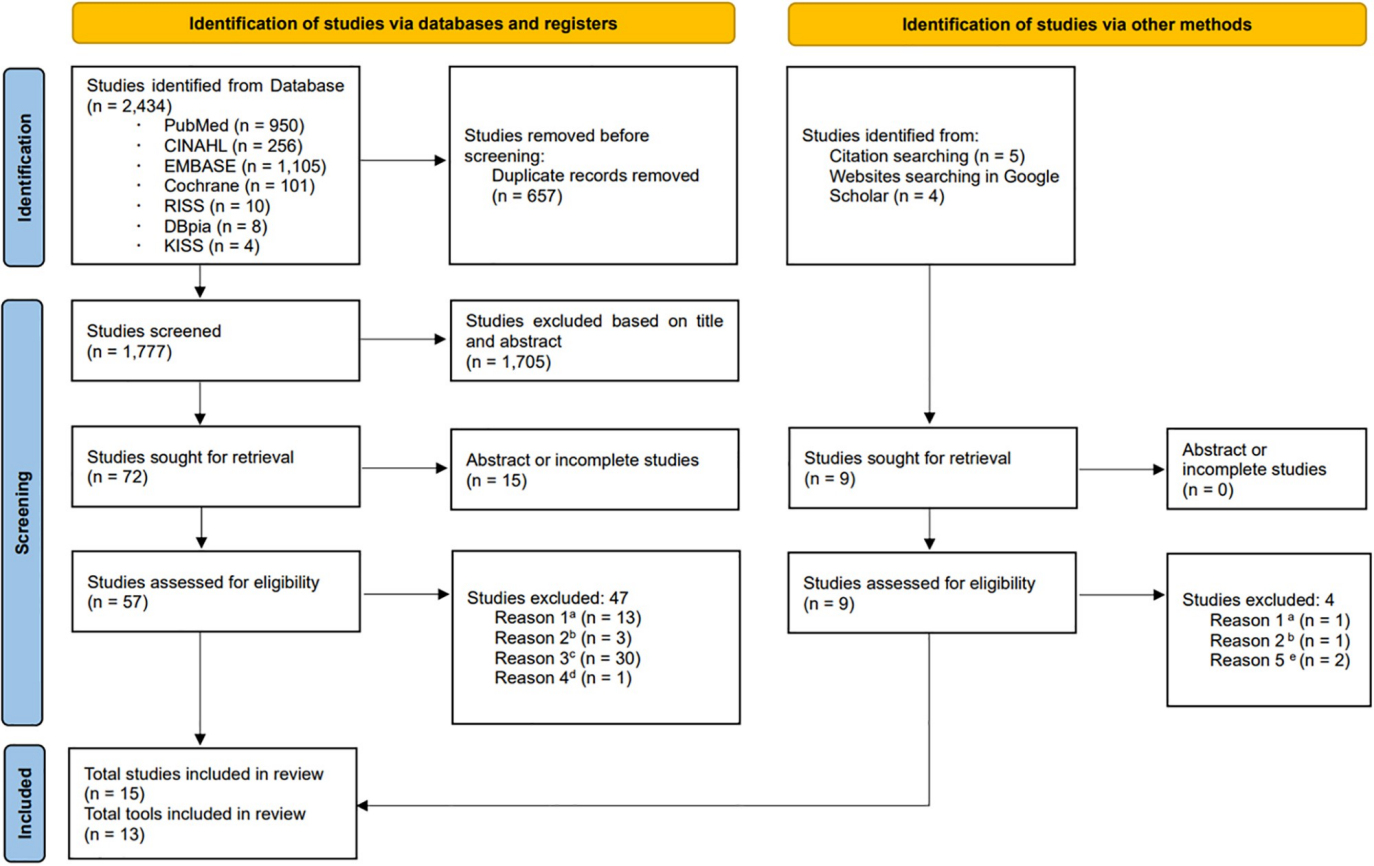
PRISMA 2020 flow chart of study selection. (a) Not a measure of knowledge of kidney transplantation. (b) Participants were not kidney transplant recipients or kidney transplant candidates. (c) No measuring tool or no description of the tool. (d) Not English or Korean. (e) Dissertation.

Of the 2,434 studies searched through the English and Korean databases, and after removing duplicated studies, 1,777 studies were retrieved. After the titles and abstracts were reviewed according to the inclusion and exclusion criteria, 72 studies were initially selected, and 10 of these were included in the final review. In addition, nine studies were retrieved from manual searches of reference lists and through Google Scholar, and five of these were included. The final analysis included 15 studies and 13 tools (Fig 1).

### Description of the included studies

Table 1 summarizes the general characteristics of the included studies. This review analyzed 15 studies. These included studies from Korea (n = 5), Canada (n = 2), Germany (n = 2), the USA (n = 2), Bangladesh (n = 1), Hungary (n = 1), the Netherlands (n = 1), and Norway (n = 1). The years of publication ranged from 2008 to 2021, with 11 (73.3%) published between 2010 and 2019. The study designs consisted of eight cross-sectional studies (53.3%), four methodological studies (26.7%), and three quasi-experimental studies (20.0%). These studies developed tools to measure KT knowledge for KT patients or candidates or analyzed the validity of existing tools. Of these, seven studies (46.7%) targeted KT patients, five (33.3%) targeted KT candidates, and three (20.0%) included both groups.

**Table 1:** General characteristics of included studies. (N = 15).

Category	Content	N (%)
Country	Bangladesh	1 (6.7)
	Canada	2 (13.3)
	Germany	2 (13.3)
	Hungary	1 (6.7)
	Korea	5 (33.3)
	Netherlands	1 (6.7)
	Norway	1 (6.7)
	USA	2 (13.3)
Published year (years)	2001–2009	1 (6.7)
	2010–2019	11 (73.3)
	≥2020	3 (20.0)
Research design	Cross-sectional study	8 (53.3)
	Methodological study	4 (26.7)
	Quasi-experimental study	3 (20.0)
Participants	KT candidates	5 (33.3)
	KT recipients	7 (46.7)
	Both	3 (20.0)

KT, kidney transplantation.

### Assessment methods of the knowledge measurement tools for KT

Table 2 presents assessment methods for the KT knowledge measurement tools used in each study. The tools consisted of a minimum of five items [14] to a maximum of 33 items [15]. Of these, six studies consisted of true-false, multiple-choice, or simple-choice questions. Most tools use true-false questions [5, 6, 11, 14–18] and multiple-choice questions [4–6, 9, 14–16, 18–20] to assess KT knowledge. Three tools used a 5-point Likert scale [8, 21, 22], with total scores ranging from 0 to 132 and higher scores indicating higher knowledge.

**Table 2:** Summary of the knowledge measurement tools for KT.

Reference (Authors)	Participants	Scale name/ Number of items	Subscales/Number of items	Scoring (Range/Total score)	Psychometrics		Strength
					Reliability	Validity	
Ismail et al. (2013)	KT recipients: 187 KT candidates: 82 General population: 1,065	R3K-T (21)	· Dialysis (8), · Kidney transplantation (3), · Living donation (10)	· True-false (16 items) · Multiple-choice question (5 items) (0-21/21)	· Cronbach’s α = .81	· Content: expert panel · Item analysis: CTT, IRT	· Reliability and validity assessed · Knowledge level can be measured
Barth et al. (2021)	KT candidates: 254	Patients’ knowledge of renal replacement therapies (15)	· Dialysis treatment (4), · Kidney transplantation (5), · Living-donor kidney transplantation (6)	· True-false · Simple-choice types of questions (0-15/15)	· N/A	· N/A	· Knowledge level can be measured · Knowledge level can be measured
de Boer et al. (2020)	KT recipients: 702	Patients’ knowledge about the ISM (8)	· The immunosuppressant knowledge questionnaire (8)	· Multiple-choice questions (0-12/12)	· N/A	· N/A	· Short measurement time · Knowledge level can be measured
Bertram et al. (2016)	KT recipients: 239	Patients’ knowledge about the ISM (8)	· The immunosuppressant knowledge questionnaire (8)	· Multiple-choice question (0-132/132)	· N/A	· N/A	· Short measurement time · Knowledge level can be measured
Iqbal et al. (2018)	KT candidates: 108 Caregivers: 40 General population: 70	KAP questionnaire (33)	· Kidney disease (9), · Kidney transplantation (10), · Attitude (6), · Perception (8)	· True-false (9 items) · Multiple-choice question (13 items) (0-69/69)	· N/A	· N/A	· Comprehensive item set covers multiple aspects of knowledge. · Knowledge level can be measured
Jones et al. (2016)	KT candidates: 41	K-TUT (22)	· Immunosuppressive medication, side effects, rejection, complication, infection & control, lifestyle, transplant terminology & others (22)	· True-false (9 items) · Multiple-choice question (13 items) (0-69/69)	· Cronbach’s α = .80	· Content: expert panel	· Comprehensive item set covers multiple aspects of knowledge · Reliability and validity assessed · Knowledge level can be measured
Rosaasen et al. (2017)	KT candidates: 41 KT recipients: 148	K-TUT (22)	· Immunosuppressive medication, side effects, organ rejection, complication, infection & control, lifestyle, transplant terminology & others (22)	· True-false (9 items) · Multiple-choice question (13 items) (0-69/69)	· Cronbach’s α = .79~.88 · κ = 60–100% · ICC = .76~.93	· Content: expert panel · Construct validity (r) = .52 (*p*< .001)	· Comprehensive item set covers multiple aspects of knowledge · Reliability and validity assessed · Knowledge level can be measured
Kang et al. (2020)	KT candidates: 29 KT recipients: 91	Korean version of K-TUT (22)	· Immunosuppressive medication, side effects, organ rejection, complication, infection & control, lifestyle, transplant terminology & others (22)	· True-false (9 items) · Multiple-choice question (13 items) (0-69/69)	· KR-20 = .89 ~ .94 for KT candidates, .76 ~ .78 for KT recipients · ICC = .91 for KT candidates, .88 for KT recipients	· Content: expert panel · Convergent validity: r = .74(*p* < .001) for KT candidates, r = .57 (*p* < .001) for KT recipients · Criterion validity: r = .31 (*p* = .003) · Item analysis: CTT	· Comprehensive item set covers multiple aspects of knowledge · Reliability and validity assessed · Knowledge level can be measured
Peipert et al. (2019)	KT candidates: 1,294	KART (15)	· Kidney function after KT, life satisfaction, conditions for KT, waiting period, period of use of the transplanted kidney (15)	· True-false (0-30/30)	· Marginal reliability = .75	· Construct validity: Cohen’s d = 0.44 ~ 0.64. · Item analysis: IRT Difficulty = -1.80 ∼ 1.65 Discrimination = 0.55 ∼ 1.21	· Comprehensive item set covers multiple aspects of knowledge · Reliability and validity assessed · Knowledge level can be measured
Reese et al. (2008)	KT candidates: 96	Knowledge about clinical outcomes with live donor kidney transplantation (5)	· Knowledge about clinical outcomes with live donor kidney transplantation (5)	· Multiple-choice question (4 items) · True-false (1 item) (0-5/5)	· Cronbach’s α > .5	· Principal components analysis	· Reliability and validity assessed · Knowledge level can be measured · Short measurement time
Urstad et al. (2011)	KT recipients: 159	Level of knowledge five days after transplant (19)	· Medication (4), · Rejection (4), · Lifestyle (11)	· 5-point Likert scale (0-19/19)	· N/A	· N/A	· Comprehensive item set covers multiple aspects of knowledge
Sim & Son (2012)	KT recipients: 42	Self-care knowledge for KT (23)	· Medication & routine check-ups (8), · Complication &prevention (8), · Exercise, weight control (2) · Diet, smoking, drinking (3) · Emotional difficulties & coping (2)	· 5-point Likert scale (23-115/115)	· Cronbach’s α = .95	· Content: expert panel	· Comprehensive item set covers multiple aspects of knowledge · Reliability and validity assessed
Hwang & Lee (2015)	KT recipients: 43	Self-care knowledge for KT (20)	· Kidney function (3), · Medication (5), · Diet (3) · Side effect & lifestyle (9)	· Multiple-choice question (0-20/20)	· Cronbach’s α = .68	· N/A	· Comprehensive item set covers multiple aspects of knowledge · Reliability assessed
Lee et al. (2019)	KT recipients: 132	Self-care knowledge for KT (20)	· Kidney function (3), · Medication (7), · Side effect (4), · Diet & body weight (3), · Activity & others (3)	· Multiple-choice question (0-100/100)	· KR-20 = .80	· N/A	· Comprehensive item set covers multiple aspects of knowledge · Reliability assessed
Jang et al. (2017)	KT recipients: 19	Medication knowledge related to KT (6)	· Medication types (1), · Medication purpose (1), · Dosage (1), · Taking time (1), · Side effects (1), · Cautions & interaction (1)	· 5-point Likert scale (6-30/30)	· N/A	· Content: expert panel	· Comprehensive item set covers multiple aspects of knowledge · Validity assessed · Short measurement time

Abbreviations: CFI, comparative fit index; CTT, classical test theory; ICC, intraclass correlation coefficient; IRT, item response theory; ISM, immunosuppressive medication; κ, kappa coefficient; KAP questionnaire, knowledge, attitude, and perception questionnaire; KART, knowledge assessment of renal transplantation; KR-20, Kuder-Richardson 20; KT, kidney transplantation; K-TUT, kidney transplant understanding tool; N/A, not applicable; R3K-T, Rotterdam renal replacement knowledge-test; RMSEA, root mean square error of approximation; S-CVI, scale-level content validity index.

### Domains of the knowledge measurement tools for KT

The domains of the KT knowledge measurement tool used in each study differ according to study participants (Table 2). For KT patients [4, 8, 9, 19–22], domains include: knowledge of immunosuppressants [4, 8, 9, 19–22], kidney function [4, 9], rejection [21], knowledge of side effects, prevention of complications after KT [4, 8, 9], lifestyle [9, 21], exercise and weight control [4, 8], diet [4, 8, 9], and smoking, drinking, emotional difficulties and coping [8]. In contrast, domains of knowledge in KT candidates [5, 11, 14–15, 17] include knowledge of kidney disease [15], dialysis [17], KT [15, 17], living-donor KT (LDKT) [17], immunosuppressants, side effects, rejection, complications, infection control, lifestyle, terminology for KT [5], kidney function after KT, life satisfaction, waiting period for KT, duration of use of the transplanted kidney [11], and the clinical outcomes of LDKT [14]. The domains of the tools used in studies with both groups (KT patients and candidates) [6, 16, 18] include knowledge of dialysis and KT, living donation [16], side effects, rejection, complications, infection control, lifestyle, and terminology for KT [6, 18].

### Validation and quality assessment of the knowledge measurement tools for KT

Table 2 presents the validation of KT knowledge measurement tools. The Rotterdam Renal Replacement Knowledge Test (R3K-T) [17], the Kidney Transplant Understanding Tool (K-TUT) [5, 6], the Korean version of K-TUT [18], Knowledge Assessment of Renal Transplantation (KART) [11], knowledge about clinical outcomes with live-donor kidney transplantation [14], and self-management knowledge for KT [8] confirmed both reliability and validity in psychometrics. The tools verified only by reliability and validity were self-management knowledge of KT [4, 9] and medication knowledge related to KT [22]. Nine studies used Cronbach’s α or the Kuder-Richardson Formula 20 (KR-20) to verify reliability. However, the K-TUT [5, 6] and the Korean version of the K-TUT [18] additionally verified their reliability using kappa and interclass correlation (ICC). Six studies used content validity to measure validity, and the K-TUT [17] additionally used factor and item analyses [5, 6]. Convergent validity, criterion validity, and item analysis were used in the Korean version of the K-TUT [18], whereas construct validity and item analysis were used in the KART [11]. Some tools used in the studies did not provide both reliability and validity of the domains, including patients’ knowledge of renal replacement therapies [17], patients’ knowledge about immunosuppressive medication (ISM) [19, 20], Knowledge, Attitudes, and Practices (KAP) questionnaire [15], and knowledge-level five days post-transplantation [21]. Of the 13 tools, 10 were available for quality assessment, 2 were rated as “adequate” or higher, 4 as “weak,” and 4 as “very weak” (Table 3).

**Table 3:** Quality appraisal using the psychometric grading framework.

Scale name	Reference	Psychometrics measures						Grade of psychometric Strength
		Content validity	Construct validity	Criterion validity	Internal consistency	Test-retest reliability	Inter-rater reliability	
R3K-T	Ismail et al. (2013)	**C**	**-** ^a^	**-**	**B**	**-**	**-**	Weak
Patients’ knowledge of renal replacement therapies	Barth et al. (2021)	**-**	**-**	**-**	**-**	**-**	**-**	N/A
Patients’ knowledge about the ISM	de Boer et al. (2020),	**-**	**-**	**-**	**-**	**-**	**-**	N/A
	Bertram et al. (2016)							
KAP questionnaire	Iqbal et al. (2018)	**-**	**-**	**-**	**-**	**-**	**-**	N/A
K-TUT	Jones et al. (2016),	**C**	**A**	**-**	**C**	**A**	**C**	Adequate
	Rosaasen et al. (2017)							
Korean version of K-TUT	Kang et al. (2020)	**C**	**A**	**B**	**C**	**A**	**-**	Good
KART	Peipert et al. (2019)	**-**	**C**	**-**	**C**	**-**	**-**	Very weak
Knowledge about clinical outcomes with live donor kidney transplantation	Reese et al. (2008)	**-**	**D**	**-**	**D**	**-**	**-**	Very weak
Level of knowledge five days after transplant	Urstad et al. (2011)	**-**	**-**	**-**	**-**	**-**	**-**	N/A
Self-care knowledge for KT	Sim & Son (2012)	**C**	**-**	**-**	**A**	**-**	**-**	Weak
Self-care knowledge for KT	Hwang & Lee (2015)	**-**	**-**	**-**	**D**	**-**	**-**	Very weak
Self-care knowledge for KT	Lee et al. (2019)	**-**	**-**	**-**	**B**	**-**	**-**	Weak
Medication knowledge related to KT	Jang et al. (2017)	**C**	**-**	**-**	**-**	**-**	**-**	Very weak

Abbreviations: ISM, immunosuppressive medication; KAP questionnaire, knowledge, attitude, and perception questionnaire; KART, knowledge assessment of renal transplantation; KT, kidney transplantation; K-TUT, kidney transplant understanding tool; N/A, not applicable; R3K-T, Rotterdam renal replacement knowledge test

^a^–was indicated unclear psychometrics measure.

## Discussion

This study aimed to identify the KT knowledge measurement tools developed for KT patients and candidates as of February 2022. It also aimed to confirm their assessment methods, domains, and validity to provide useful information for KT education.

Post-transplant management of KT is complicated and requires considerable knowledge [21]. It is essential to know the types and methods of immunosuppressant administration, graft monitoring, lifestyle changes, infection prevention and management, and monitoring of any signs and symptoms of rejection [6, 23]. To achieve this, knowledge must be acquired through prior education [23, 24]. Rosaasen and Mainra [7] reported that medication, KT processes, lifestyle changes post-transplantation, and infection control methods should be known before KT as these can reduce morbidity, mortality, and the cost of health and disease management [25]. Insufficient knowledge can lead to serious consequences, such as the rejection of the transplanted kidney [6]. Therefore, providing appropriate education based on the results of the knowledge evaluation of patients is important throughout the transplantation process [6].

The literature analysis confirmed that most KT knowledge measurement tools have been developed in Korea. Since they were developed for KT patients, there were limitations such as “Guidelines for outpatient visits after KT [4, 8, 9],” which are inapplicable to both KT patients and candidates. However, there are three tools for both KT patients and KT candidates: the R3K-T [16], K-TUT [6], and the Korean version of K-TUT [18]. The Korean version of the K-TUT has verified its reliability and validity and can be applied to Korean KT patients and candidates.

### Assessment methods of the knowledge measurement tools for KT

Some previous studies have measured KT knowledge using simple questions, instead of validated tools [26–29]. Such questions cannot accurately evaluate knowledge levels of a health condition as complex as KT, and structured tools are more suited for this purpose. Furthermore, the methods of investigating patients’ and candidates’ knowledge is also important. This review found that the number of items in the included tools ranged from five to 33, and most consisted of true-false or multiple-choice questions. To evaluate accurate knowledge, true-false, simple-choice, and multiple-choice questions are more appropriate than Likert scales. Nevertheless, none of the studies established a cutoff point that determined the level of knowledge. Livingston and Zieky [30] defined “setting the achievement level” as not finding an “existing answer,” but “determine what score was enough to be qualified” by consensus among experts. Therefore, in future studies, it is necessary to set a cutoff point using receiver operator characteristic (ROC) curve analysis to determine an appropriate level of KT knowledge.

### Domains of the knowledge measurement tools for KT

The KT knowledge measurement tools had different evaluation domains depending on the participants. The tools for KT patients mainly evaluate knowledge of immunosuppressants, renal function, rejection, complications, and lifestyles, such as exercise, weight control, diet, smoking, and alcohol consumption. Among the tools for KT patients, some domains measured immunosuppressant-related knowledge and could be applied to KT candidates as prior learning. These include patients’ knowledge about ISM [19, 20] and medication knowledge related to KT [22]. A caveat of this recommendation is that medication knowledge related to KT [22] was measured using a 5-point Likert scale and was difficult to accurately assess. For this recommendation to be effective, it is necessary to select an appropriate tool and form of measurement.

The tools for KT candidates included some knowledge assessment that is applicable only to candidates, such as knowledge of dialysis and the waiting period, patients’ knowledge of renal replacement therapies [17] and R3K-T [16], including dialysis. Hence, tools for KT candidates are limited to be used as tools for measuring KT knowledge.

### Validation and quality assessment of the knowledge measurement tools for KT

Internal consistency reliability was the most frequently used reliability of the 13 tools. It evaluates the similarity of items on a scale and tends to increase as the number of items increases. Additional reliability measures would be beneficial. For instance, the K-TUT [6, 18] and the Korean version of the K-TUT [18] were evaluated for test-retest and inter-rater reliability, indicating agreement and consistency with the evaluation results. The inter-rater reliability of the K-TUT [6, 18], measured using the kappa coefficient, was 60–100%. To increase reliability, raters must understand how to use the tool in advance [31]. Other tools with unclear reliability measures [15, 17, 19–22] are difficult to consider reliable. In addition, tools that only had internal consistency [4, 8, 9, 14, 16] required effort to further increase their agreement and consistency.

Most tools were also evaluated for and held content validity, such as those tools developed in Korea [8, 23]. Others [4, 9, 15, 17, 19, 20] were insufficient in evaluating validity because they did not provide content validity. To measure this, factor analysis is used to understand tools’ complex structures by simplifying the items into several factors. Both the R3K-T and KART tools were used to evaluate construct validity.

On the other hand, convergent validity is used to evaluate whether measurement results are similar or correlated between tools measuring the same concept [32], while criterion validity evaluates how well the measurement results of the tool reflect or predict the gold standard [32]. The Korean version of the K-TUT had both convergent and criterion validity.

Ensuring reliability and validity of tools is important in order to avoid different interpretations or measurements between raters [33]. In this study, the PGF was used to evaluate the quality of the tools, and only two tools showed a grade of “adequate” or higher, while most showed “weak” or “very weak.” These results were similar to those of a systematic review of evidence-based knowledge measurement tools in nursing practice [34] and a systematic review of tools for measuring learning outcomes in healthcare students using the PGF [35]. This means that there is not a wide range of high-quality tools to select from. Rigorous validations and reliability tests are required after tool development to increase their quality and usefulness.

### Clinical implications

Knowledge measurement tools with high validity can be utilised to evaluate the effectiveness of educational interventions in improving self-management after KT. Improving the candidates’ knowledge of KT can reduce their fear and help them prepare for their transplants. In addition, since prognosis can be improved by receiving education in advance, these tools can evaluate the effectiveness of such educational interventions specifically for KT candidates. However, since the tools for KT candidates include multiple domains such as knowledge of dialysis or donation, careful and domain-specific selection is required when aiming to measure KT knowledge or self-management effectiveness. Alternatively, separate tools for KT patients and KT candidates may be more appropriate. These can also make clearer whether educational interventions pre-transplant help improve patients’ self-management post-transplant.

### Limitations

Despite its various advantages, this study had several limitations. Firstly, it only included KT knowledge measurement tools for KT patients and candidates. Therefore, these tools are limited to evaluating nurses, medical students, and nursing students with medical knowledge. Secondly, the tools reviewed had different scoring systems which may have led to inconsistent results across studies, making it difficult to compare these. Thirdly, since this study evaluated only studies published in English or Korean, the representativeness of our results may be reduced. Future research should aim to compare and analyze studies published in languages other than English and Korean.

Additionally, knowledge level does not necessarily translate into health behavior. Therefore, future studies should measure health outcomes based on knowledge level. The PGF used for the quality assessment of the tools also did not include responsiveness, which is an important aspect of validity. This is often omitted from early feasibility studies because it requires longitudinal data and time to test the instrument in this capacity. Therefore, future studies should utilize an assessment tool that includes responsiveness. The tools required for KT thus require rigorous validation both in development and in their use overtime, and they should enable researchers to measure their effectiveness against established health outcomes.

## Conclusions

This integrated literature review was conducted to identify a knowledge-level evaluation tool for KT, applicable to both KT patients and candidates, and provide an overview of its evaluation method and validity. Among the various tools investigated, the K-TUT and the Korean version of the K-TUT were used to measure the knowledge level of various aspects of KT. They were confirmed to be reliable and valid tools, however, their reliability and validity has not yet been demonstrated. This is necessary gap for future research to bridge, along with the need to empirically determine KT knowledge levels (such as by using ROC curve analysis) and to set health outcomes that can aid in evaluating the effectiveness of educational interventions to promote self-management after KT.

## Supporting information

S1 Table PRISMA 2020 abstract checklist. (PDF)

S2 Table PRISMA 2020 checklist. (PDF)

S1 File International prospective register of systematic review. (PDF)
