# Supplementary material for: An integrative literature review of kidney transplantation knowledge tools
Source: PLoS One. 2023 Jan 31;18(1):e0281073. doi: 10.1371/journal.pone.0281073 (PMC9888680; doi:10.1371/journal.pone.0281073)
Supplement: S1 File — (PDF) [file pone.0281073.s003.pdf]

NHS
National Institute for Health Research

UNIVERSITY of York
Centre for Reviews and Dissemination

Please note the PROSPERO will be unavailable from 09:00 BST on Saturday 15th October to 09:00 BST on Monday 17th October for essential maintenance

---

Systematic review

A list of fields that can be edited in an update can be found here

1. * Review title.

Give the title of the review in English

An Systematic Integrative Literature Review of Kidney Transplantation Knowledge Tools

2. Original language title.

For reviews in languages other than English, give the title in the original language. This will be displayed with the English language title.

An Systematic Integrative Literature Review of Kidney Transplantation Knowledge Tools

3. * Anticipated or actual start date.

Give the date the systematic review started or is expected to start.

10/02/2022

4. * Anticipated completion date. [1 change]

Give the date by which the review is expected to be completed.

11/03/2022

5. * Stage of review at time of this submission. [1 change]

This field uses answers to initial screening questions. It cannot be edited until after registration.

Tick the boxes to show which review tasks have been started and which have been completed.

Update this field each time any amendments are made to a published record.

The review has not yet started: No

Review stage	Started	Completed
Preliminary searches	Yes	Yes
Piloting of the study selection process	Yes	Yes
Formal screening of search results against eligibility criteria	Yes	Yes
Data extraction	Yes	Yes
Risk of bias (quality) assessment	Yes	Yes
Data analysis	Yes	Yes

Provide any other relevant information about the stage of the review here.

6. * Named contact.

The named contact is the guarantor for the accuracy of the information in the register record. This may be any member of the review team.

Chan Mi Kang

Email salutation (e.g. "Dr Smith" or "Joanne") for correspondence:

Dr Kang

7. * Named contact email.

Give the electronic email address of the named contact.

chan-mi0701@hanmail.net

8. Named contact address

Give the full institutional/organisational postal address for the named contact.

Department of Nursing, Dong-Eui Institute of Technology, 54, Yangji-ro, Busanjin-gu, Busan, South Korea

9. Named contact phone number.

Give the telephone number for the named contact, including international dialling code.

821085841446

10. * Organisational affiliation of the review.

Full title of the organisational affiliations for this review and website address if available. This field may be completed as 'None' if the review is not affiliated to any organisation.

Dong-Eui Institute of Technology

Organisation web address:

11. * Review team members and their organisational affiliations. [1 change]

Give the personal details and the organisational affiliations of each member of the review team. Affiliation refers to groups or organisations to which review team members belong. **NOTE: email and country now MUST be entered for each person, unless you are amending a published record.**

Dr Chan Mi Kang. Dong-Eui Institute of Technology
Dr Hyejin Lee. Department of Nursing, Dong-Eui University

12. * Funding sources/sponsors.

Details of the individuals, organizations, groups, companies or other legal entities who have funded or sponsored the review.

none

Grant number(s)

State the funder, grant or award number and the date of award

13. * Conflicts of interest.

List actual or perceived conflicts of interest (financial or academic).

None

14. Collaborators.

Give the name and affiliation of any individuals or organisations who are working on the review but who are not listed as review team members. **NOTE: email and country must be completed for each person, unless you are amending a published record.**

15. * Review question.

State the review question(s) clearly and precisely. It may be appropriate to break very broad questions down into a series of related more specific questions. Questions may be framed or refined using PI(E)COS or similar where relevant.

(1) What were the measurement methods for evaluating kidney transplantation knowledge between KT patients and candidates?

(2) What knowledge domains the tools consisted of?

(3) How were the measurement tools verified?

16. * Searches.

State the sources that will be searched (e.g. Medline). Give the search dates, and any restrictions (e.g. language or publication date). Do NOT enter the full search strategy (it may be provided as a link or attachment below.)

We will search four English databases (MEDLINE via PubMed, EMBASE, CINAHL, and Cochrane Library) and three Korean databases (Research Information Sharing Service [RISS], Korean Studies Information Service System [KISS], and Data Base Periodical Information Academic [DBpia]).

17. URL to search strategy.

Upload a file with your search strategy, or an example of a search strategy for a specific database, (including the keywords) in pdf or word format. In doing so you are consenting to the file being made publicly accessible. Or provide a URL or link to the strategy. Do NOT provide links to your search **results**.

Medical Subject Headings (MeSH) will be used to select keywords, and frequent keywords were identified based on a review of the English titles and keywords.

Search strategy: (“kidney transplant*” OR “renal transplant*” OR “kidney replace*” OR “renal replace*” OR “kidney graft” OR “renal graft”) AND (“knowledge” OR “awareness”) AND (“scale” OR “tool” OR “instrument” OR “questionnaire” OR “inventory”).

Alternatively, upload your search strategy to CRD in pdf format. Please note that by doing so you are consenting to the file being made publicly accessible.

Do not make this file publicly available until the review is complete

18. * Condition or domain being studied.

Give a short description of the disease, condition or healthcare domain being studied in your systematic review.

Chronic renal failure

19. * Participants/population.

Specify the participants or populations being studied in the review. The preferred format includes details of both inclusion and exclusion criteria.

Kidney transplantation patients, Kidney transplantation candidates

20. * Intervention(s), exposure(s).

Give full and clear descriptions or definitions of the interventions or the exposures to be reviewed. The preferred format includes details of both inclusion and exclusion criteria.

The inclusion criteria : 1) studies that focused on knowledge of kidney transplantation, 2) subjects that

included kidney transplantation patients or candidates (kidney disease, renal failure, peritoneal dialysis, or hemodialysis patients), 3) studies that were complete reports or had full-text available, and 4) were written in Korean or English. The exclusion criteria : 1) studies with no explanation of the tools or 2) dissertations, editorials, conference abstracts, or review papers.

21. * Comparator(s)/control.

Where relevant, give details of the alternatives against which the intervention/exposure will be compared (e.g. another intervention or a non-exposed control group). The preferred format includes details of both inclusion and exclusion criteria.

Not applicable

22. * Types of study to be included.

Give details of the study designs (e.g. RCT) that are eligible for inclusion in the review. The preferred format includes both inclusion and exclusion criteria. If there are no restrictions on the types of study, this should be stated.

We will include studies that were complete reports or had full-text available but we will exclude dissertations, editorials, conference abstracts, or review papers.

23. Context.

Give summary details of the setting or other relevant characteristics, which help define the inclusion or exclusion criteria.

24. * Main outcome(s).

Give the pre-specified main (most important) outcomes of the review, including details of how the outcome is defined and measured and when these measurement are made, if these are part of the review inclusion criteria.

Knowledge of KT was positively correlated with self-management performance and treatment compliance post-transplantation (Lee et al., 2019; Sim & son 2012), and was important for the transplanted kidney to be well-maintained (Jones et al., 2016). Even for KT candidates, knowledge regarding KT and post-transplant management can reduce fear and confusion (Rosaasen et al., 2017) and help them adapt to the changes in their healthcare and lifestyle post-transplantation. Therefore, tools that accurately assess the knowledge level of KT are required for nurses to provide appropriate education and information to KT patients and candidates.

Various studies have evaluated the level of knowledge regarding kidney transplantation(KT) and provided related interventions for KT patients or candidates. These were mainly intervention studies that consisted of education and telephone counseling (Hwang & Lee, 2015), a survey on the knowledge and treatment adherence of KT patients (Kim, 1995), and a psychometric test of the knowledge measurement tools for KT (Peipert et al., 2019). In contrast, it was difficult to find a study that comprehensively analyzed the kinds of

knowledge measurement tools used in these intervention studies and their reliability and validity.

Measures of effect

Please specify the effect measure(s) for you main outcome(s) e.g. relative risks, odds ratios, risk difference, and/or 'number needed to treat.

Assessment Methods of the Knowledge Measurement Tools for KT, Number of items, Subscales, Scoring, Reliability(Cronbach's a, Kappa, ICC, KR-20), Vailidity(Content validity, construct validity, vonvergent validity, criteron validity, item analysis)

25. * Additional outcome(s).

List the pre-specified additional outcomes of the review, with a similar level of detail to that required for main outcomes. Where there are no additional outcomes please state 'None' or 'Not applicable' as appropriate to the review

None

Measures of effect

Please specify the effect measure(s) for you additional outcome(s) e.g. relative risks, odds ratios, risk difference, and/or 'number needed to treat.

26. * Data extraction (selection and coding).

Describe how studies will be selected for inclusion. State what data will be extracted or obtained. State how this will be done and recorded.

Literature will be selected by two independent researchers, and any disagreements and opinions were solved through sufficient discussion at a meeting.). The retrieved literature will be listed, reviewed, and organized using a reference management software (EndNote 20.2.1). After duplicate studies will be removed, the titles, abstracts, and full texts will be reviewed in stages according to the inclusion and exclusion criteria. The final studies will be selected through a meeting among the researchers.

27. * Risk of bias (quality) assessment.

State which characteristics of the studies will be assessed and/or any formal risk of bias/quality assessment tools that will be used.

The quality of the measurement tools will evaluate independently by two reviewers using the psychometric grading framework (PGF). The PGF is based on the most commonly used statistical tests and values recommended by leading psychologists and biostatisticians. It is evaluated from A to D according to the psychological measures used in the tool: content, construct, and criterion validities, internal consistency, and test-retest and inter-rater reliabilities. Subsequently, the overall psychometric strength was evaluated by combining the measured number of psychometrics and the levels from A to D. Three or more As and/or Bs were rated as good, two As and/or Bs ± C or D as adequate, one A or B ± C or D as weak, and one or more

Ds as weak. Literature will be selected by two independent researchers, and any disagreements and opinions will be solved through sufficient discussion at a meeting.

28. * Strategy for data synthesis.

Describe the methods you plan to use to synthesise data. This **must not be generic text** but should be **specific to your review** and describe how the proposed approach will be applied to your data. If meta-analysis is planned, describe the models to be used, methods to explore statistical heterogeneity, and software package to be used.

The general characteristics of the included studies will be analyzed in the following order: country, publication year, research design, and study subjects. The knowledge tools for kidney transplantation will be analyzed in the following order: applied subjects, scale names, total number of items, domains, score calculation method, score range, total score, and psychometrics, which included reliability and validity. Two researchers will independently review and integrate the entire literature, and sufficient discussions and reviews were repeated until a common opinion was drawn on the discrepancy items.

29. * Analysis of subgroups or subsets.

State any planned investigation of ‘subgroups’. Be clear and specific about which type of study or participant will be included in each group or covariate investigated. State the planned analytic approach.

The quality of the measurement tools will evaluate independently by two reviewers using the psychometric grading framework (PGF). The PGF is based on the most commonly used statistical tests and values recommended by leading psychologists and biostatisticians. It is evaluated from A to D according to the psychological measures used in the tool: content, construct, and criterion validities, internal consistency, and test-retest and inter-rater reliabilities. Subsequently, the overall psychometric strength was evaluated by combining the measured number of psychometrics and the levels from A to D. Three or more As and/or Bs were rated as good, two As and/or Bs ± C or D as adequate, one A or B ± C or D as weak, and one or more Ds as weak. Literature will be selected by two independent researchers, and any disagreements and opinions will be solved through sufficient discussion at a meeting.

30. * Type and method of review.

Select the type of review, review method and health area from the lists below.

Type of review

Cost effectiveness
No

Diagnostic
No

Epidemiologic
No

Individual patient data (IPD) meta-analysis

No

Intervention

No

Living systematic review

No

Meta-analysis

No

Methodology

No

Narrative synthesis

No

Network meta-analysis

No

Pre-clinical

No

Prevention

No

Prognostic

No

Prospective meta-analysis (PMA)

No

Review of reviews

No

Service delivery

No

Synthesis of qualitative studies

No

Systematic review

Yes

Other

Yes

A systematic integrative literature review of tools

Health area of the review

Alcohol/substance misuse/abuse

No

Blood and immune system

No

Cancer

No

Cardiovascular

No

Care of the elderly

No

Child health

No

Complementary therapies

No

COVID-19

No

Crime and justice

No

Dental

No

Digestive system

No

Ear, nose and throat

No

Education

No

Endocrine and metabolic disorders

No

Eye disorders

No

General interest

No

Genetics

No

Health inequalities/health equity

No

Infections and infestations

No

International development

No

Mental health and behavioural conditions

No

Musculoskeletal

No

Neurological

No

Nursing

Yes

Obstetrics and gynaecology

No

Oral health

No

Palliative care

No

Perioperative care

No

Physiotherapy

No

Pregnancy and childbirth

No

Public health (including social determinants of health)

No

Rehabilitation

No

Respiratory disorders

No

Service delivery

No

Skin disorders

No

Social care

No

Surgery

No

Tropical Medicine

No

Urological

Yes

Wounds, injuries and accidents

No

Violence and abuse

No

31. Language.

Select each language individually to add it to the list below, use the bin icon to remove any added in error.

English

There is not an English language summary

32. * Country.

Select the country in which the review is being carried out. For multi-national collaborations select all the countries involved.

South Korea

33. Other registration details.

Name any other organisation where the systematic review title or protocol is registered (e.g. Campbell, or The Joanna Briggs Institute) together with any unique identification number assigned by them. If extracted data will be stored and made available through a repository such as the Systematic Review Data Repository (SRDR), details and a link should be included here. If none, leave blank.

34. Reference and/or URL for published protocol.

If the protocol for this review is published provide details (authors, title and journal details, preferably in Vancouver format)

Whittemore R, Knafl K. The integrative review: updated methodology. J. Adv. Nurs. 2005;52(5):546-553.

doi:10.1111/j.1365-2648.2005.03621.x

Add web link to the published protocol.

Or, upload your published protocol here in pdf format. Note that the upload will be publicly accessible.

No I do not make this file publicly available until the review is complete

Please note that the information required in the PROSPERO registration form must be completed in full even if access to a protocol is given.

35. Dissemination plans.

Do you intend to publish the review on completion?

Yes

Give brief details of plans for communicating review findings.?

36. Keywords.

Give words or phrases that best describe the review. Separate keywords with a semicolon or new line. Keywords help PROSPERO users find your review (keywords do not appear in the public record but are included in searches). Be as specific and precise as possible. Avoid acronyms and abbreviations unless these are in wide use.

kidney transplantation; knowledge; review; literature review; psychometrics

37. Details of any existing review of the same topic by the same authors.

If you are registering an update of an existing review give details of the earlier versions and include a full bibliographic reference, if available.

38. * Current review status. [1 change]

Update review status when the review is completed and when it is published.New registrations must be ongoing so this field is not editable for initial submission.

Please provide anticipated publication date

Review_Completed_not_published

39. Any additional information.

Provide any other information relevant to the registration of this review.

40. Details of final report/publication(s) or preprints if available.

Leave empty until publication details are available OR you have a link to a preprint (NOTE: this field is not editable for initial submission). List authors, title and journal details preferably in Vancouver format.

Give the link to the published review or preprint.
